# Global lysine methylome profiling using systematically characterized affinity reagents

**DOI:** 10.1038/s41598-022-27175-x

**Published:** 2023-01-07

**Authors:** Christine A. Berryhill, Jocelyne N. Hanquier, Emma H. Doud, Eric Cordeiro-Spinetti, Bradley M. Dickson, Scott B. Rothbart, Amber L. Mosley, Evan M. Cornett

**Affiliations:** 1 Department of Biochemistry and Molecular Biology, Indiana University School of Medicine, Indianapolis, IN 46202 USA; 2 Department of Epigenetics, Van Andel Institute, Grand Rapids, MI 49503 USA

**Keywords:** Methylases, Biochemistry, Chemical modification, Proteomic analysis

## Abstract

Lysine methylation modulates the function of histone and non-histone proteins, and the enzymes that add or remove lysine methylation—lysine methyltransferases (KMTs) and lysine demethylases (KDMs), respectively—are frequently mutated and dysregulated in human diseases. Identification of lysine methylation sites proteome-wide has been a critical barrier to identifying the non-histone substrates of KMTs and KDMs and for studying functions of non-histone lysine methylation. Detection of lysine methylation by mass spectrometry (MS) typically relies on the enrichment of methylated peptides by pan-methyllysine antibodies. In this study, we use peptide microarrays to show that pan-methyllysine antibodies have sequence bias, and we evaluate how the differential selectivity of these reagents impacts the detection of methylated peptides in MS-based workflows. We discovered that most commercially available pan-Kme antibodies have an in vitro sequence bias, and multiple enrichment approaches provide the most comprehensive coverage of the lysine methylome. Overall, global lysine methylation proteomics with multiple characterized pan-methyllysine antibodies resulted in the detection of 5089 lysine methylation sites on 2751 proteins from two human cell lines, nearly doubling the number of reported lysine methylation sites in the human proteome.

## Introduction

Lysine methylation is a widespread, dynamic post-translational modification that regulates protein activity, localization, and molecular interactions^1,2^. Lysine methylation occurs as three distinct methyl states, corresponding to the addition of one (mono-methyl; Kme1), two (di-methyl; Kme2), or three (tri-methyl; Kme3) methyl groups to the ε-amine of lysine. Dysregulation of the enzymes that add (lysine methyltransferases; KMTs) and remove (lysine demethylases; KDMs) this dynamic modification in a variety of human diseases has spurred significant interest in studying lysine methylation^3–7^. While early studies focused almost exclusively on histone lysine methylation, studies over the last decade have detected lysine methylation on thousands of human proteins^8^, and work to study the function of these modifications remains an active area of research.

Significant efforts have been made to develop robust mass spectrometry (MS)-based methods to map hundreds of methylated lysines in a site and methyl-state specific manner across the entire proteome^9–18^. Enriching for lysine methylation has traditionally been considered a necessary step in these workflows; the most common strategy involves enriching methylated peptides or proteins by immunoprecipitation using affinity reagents designed to bind to a specific methyl state indiscriminately from the surrounding protein sequence—so-called pan-methyllysine antibodies. Similarly, methyl-reader domains have been repurposed for enrichment. This latter approach is attractive for its ability to incorporate negative control IPs using mutant proteins that lack the ability to bind lysine methylation^10,19^. The enrichment step is a key limitation of all these approaches, as the affinity reagents often exhibit a bias for specific residues surrounding the methylated lysine. In general, lysine methylation does not significantly change the physiochemical properties of the lysine side chain^20^. Each additional methyl group increases the mass by 14 Daltons and slightly increases the hydrophobicity but does not alter the charge of the side chain—representing a challenging target for selective molecular recognition. Natural readers of lysine methylation, highlighted by 2 decades of research on chromatin readers, frequently contain multiple regulatory domains resulting in multivalent recognition of modified proteoforms^21–24^. The combination of multiple domains leads to a higher affinity interaction that may not be feasible using antibodies or isolated protein domains designed to recognize a modified form of lysine regardless of the surrounding amino acid sequence. Indeed, antibodies used for mapping chromatin modifications are well documented to be plagued by selectivity issues^24–26^, which must be mitigated by careful characterization and validation prior to use. To date, no study has systematically characterized the selectivity of pan-methyllysine antibodies.

As technological advances in MS continue, a trend has been to avoid these enrichment steps. A recent study reanalyzed publicly available proteomics datasets from human cell lines that were fractionated to generate deep proteomic coverage^27^. By including lysine methylation as a potential modification in database searches, hundreds of novel lysine methylation sites were identified, suggesting it may be possible to eliminate enrichment steps. However, to our knowledge, no study has directly compared the performance of enrichment to deep fractionation without enrichment.

To address these challenges, we profiled the selectivity of commercially available pan-methyllysine antibodies using peptide microarrays. Notably, we revealed that most of these reagents exhibit sequence bias. We then hypothesized that less biased reagents would perform better for the enrichment of methylated peptides prior to analysis by MS. To test this hypothesis, we generated comparative global profiles of lysine methylation from two cell lines after offline fractionation using two antibodies specific for Kme2 and Kme3 that exhibit different degrees of sequence bias. A portion of each unenriched sample was also analyzed by LC–MS/MS. Overall, we identified over 4000 new lysine methylation sites, nearly doubling the total number of observed sites of lysine methylation reported in the PhosphoSitePlus repository for post-translational modifications^28^. Our results have important implications for future use of pan-methyllysine antibodies and suggest a combination of enrichment and deep fractionation methods lead to the identification of unique lysine methylation sites. This study represents the most detailed proteome-wide analysis of lysine methylation in human cells to date.

## Results

### Pan-methyllysine antibodies have sequence bias

Pan-methyllysine antibodies are essential reagents for lysine methylation research; these reagents are frequently used in experiments ranging from low-throughput detection of protein methylation by immunoblotting to high-throughput detection of hundreds of sites by MS. Previous studies have performed limited analyses of the sequence and methyl-state selectivity of a subset of these reagents, revealing biases that could ultimately impact the interpretation of downstream results^29^. However, no systematic characterization has ever been performed. To address this, we first used a well-established histone peptide microarray platform to determine the selectivity of these critical reagents^25,26,30,31^. Histone peptide microarrays enable high-throughput characterization of histone PTM-specific antibodies, chromatin readers, and protein modifying enzymes towards hundreds of peptides in a single experiment. In the iteration used in this study, each microarray contained approximately 260 uniquely modified peptides, including single and combinatorial modifications on the core and variant histone proteins, as well as some non-histone proteins.

Modifications included mono-, di-, and trimethylation on lysine, mono-, symmetrical di-, and asymmetrical di-methylation on arginine, phosphorylation, and acetylation. We reasoned the diversity of modification state (mono-, di-, or tri-methyl) and sequence surrounding the peptides on these microarrays would allow us to probe the selectivity of pan-methyllysine antibodies. In total, ten antibodies were tested: one pan-Kme1 antibody, four pan-Kme2 antibodies, and five pan-Kme3 antibodies (Supplementary Table 1).

**Figure 1:**
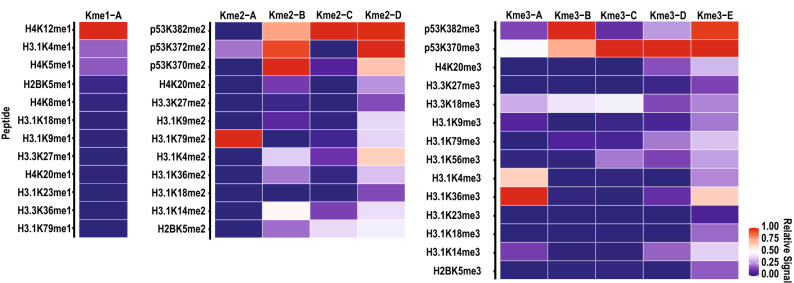
Analysis of pan-methyllysine antibodies by peptide microarrays. Heatmaps depict the average fluorescent signal intensity (n = 9) from a histone peptide microarray hybridized with a pan-methyllysine antibody. Data is normalized to the highest signal in each column on the microarray and the color code is proportional to the fluorescent signal, where red (1) indicates more antibody hybridization and blue (0) indicates less antibody hybridization. Rows show the identity of each histone peptide and columns show the pan-methyllysine antibody used.

To evaluate the sequence selectivity of these pan-methyllysine antibodies, we focused our analysis on microarray features containing only the modification each antibody is designed to recognize. For each methyl-state, this analysis was limited to approximately ten unique sequences. None of the antibodies reacted with all peptides equally (Fig. 1). Instead, most of the antibodies displayed strong preferences and were bound to less than half of the peptides containing the target methyllysine modification. Due to the striking sequence bias observed in this limited analysis, we sought to characterize the sequence selectivity of pan-methyllysine antibodies beyond the limited sequences represented on the histone peptide microarray.

We previously described a method to determine the substrate selectivity of KMTs using a lysine-oriented peptide library (K-OPL) containing an unmodified central lysine^32^, and more recently used mono-, di-, and tri-methylated K-OPLs to determine the selectivity of methyllysine reader domains and modification specific histone antibodies^33^. The K-OPL consists of 114 sets of 9-mer peptides oriented around a fixed central lysine. The sets are split into six position groups based on the location of an additional fixed amino acid within three amino acids on either side of the central lysine (designated P−3, P−2, P−1, P+1, P+2, and P+3). Each position group contains 19 sets of 9-mers, one set for each amino acid (excluding cysteine) fixed at that position. The remaining positions within each set are degenerate, resulting in an equal mixture of all other possible sequence variants. To characterize the selectivity of pan-methyllysine antibodies, we fabricated new microarrays containing unmodified, mono-, di-, and trimethyl lysine-oriented peptide libraries (Supplementary Fig. 1).

**Figure 2:**
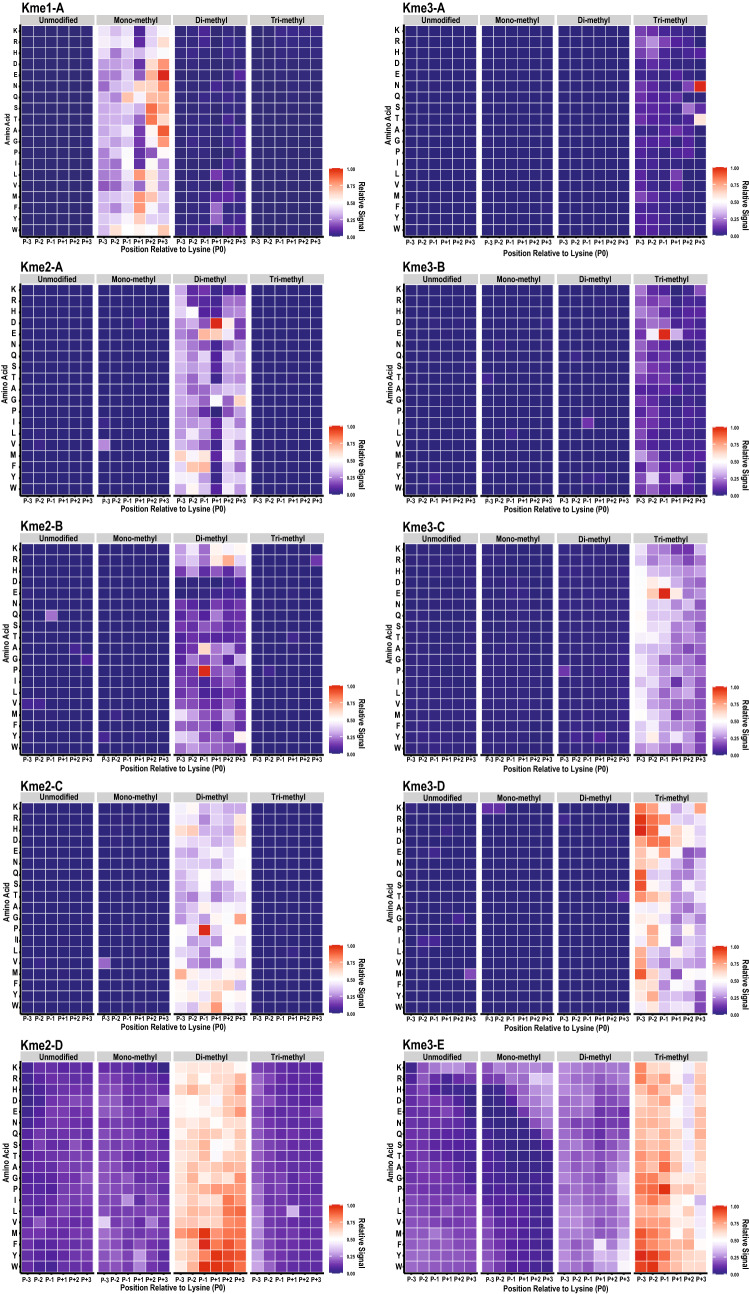
Pan-methyllysine antibodies exhibit sequence bias. Heatmaps depicting average fluorescent signal intensity (n = 3) from a microarray hybridized with the indicated commercially available antibody. Data is globally normalized to the highest signal on the microarray and the color code is proportional to the fluorescent signal, where red (1) indicates more antibody hybridization and blue (0) indicates less antibody hybridization. Rows show the identity of each fixed residue and columns show the position within the sequence relative to the central lysine residue (P0). The modification state of the central lysine residue is indicated by the title above each grouped heatmap.

Qualitative analysis of the pan-methyllysine antibodies by K-OPL microarray showed similar trends as the histone peptide microarray analysis (Fig. 2). Few antibodies strongly reacted with off-target methyl-states. However, it is notable that some antibodies weakly reacted with off-target methyl-state sets that contain the most favorable on-target amino acids. For example, the Kme1-A antibody bound strongly to mono-methyl K-OPL sets with M, F, Y, or L in the P-1 position (Fig. 2); however, it also weakly recognized those same amino acids in the di-methyl K-OPL array.

The biased sequence selectivity trend observed by histone peptide microarray analysis was also seen in the K-OPL array analysis for the Kme2 and Kme3 antibodies. Overall, Kme2-D and Kme3-E displayed higher binding to peptides in multiple sequence contexts when compared to the other pan-methyl antibodies (Fig. 2). While the overall qualitative trends were similar, specific sequence biases in the K-OPL analysis did not align with the sequence preference we observed by histone peptide arrays. For example, Kme1-A had a strong preference for H4K12me1, but the K-OPL array did not show a strong preference for any amino acids surrounding H4K12. Furthermore, Kme1-A preferred amino acid sequences present in some of the other mono-methylated peptides on the histone peptide array, such as H4K20me1 and H3.1K18me1. Almost all the antibodies showed a high signal for peptides on the histone peptide array that contained unfavorable amino acids according to the K-OPL array data. The differences seen may be explained by the nature of the assays: the K-OPL screen tests the preference for a single amino acid in a single position of an unbiased complex peptide mixture; in contrast, the histone peptide microarray tests the preference for a specific peptide sequence.

To quantitatively evaluate and compare the pan-selectivity of each antibody, we calculated a pan metric score (0–100) based on the average signal across all sets of the K-OPL with the target methyl-state. Higher pan metric scores reflect antibodies with less sequence bias. Most antibodies have a pan metric score under 50, demonstrating a bias for specific amino acids surrounding a lysine (Supplementary Fig. 2a). Antibody screening was performed with an equal amount of each antibody, but a wide range of maximum signal intensity was observed. Array signal intensity weakly correlated with the pan metric score (Supplementary Fig. 2b), suggesting that antibodies with broader sequence preference may bind with higher overall affinity to the lysine modification site. Interestingly, Kme2-A and Kme3-A are the antibodies most utilized in the literature; however, they had some of the lowest pan metric scores determined by K-OPL. Conversely, the least biased anti-Kme2 and anti-Kme3 antibodies, Kme2-D and Kme3-E, have not been used to enrich methylated peptides prior to MS analysis.

### Comparing enrichment strategies for global detection of lysine methylation by MS

**Figure 3:**
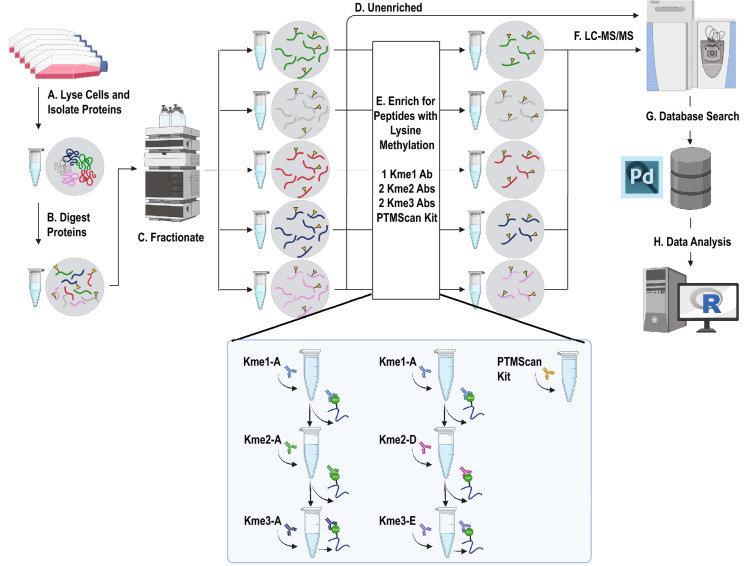
Proteomics workflow for profiling the global lysine methylome from cultured cells. (**a**) Cells are lysed in urea lysis buffer to extract proteins. (**b**) Proteins are denatured and digested to generate peptides. (**c**) Peptides are fractionated offline using high pH reversed-phase chromatography followed by fraction concatenation. (**d**) A portion of each fraction is removed for analysis without enrichment. (**e**) Methylated peptides are enriched using immunoprecipitation with pan-methyllysine antibodies. (**f**) Each enriched and unenriched fraction is analyzed using liquid chromatography-tandem mass spectrometry (LC–MS/MS). (**g**) Database searches are performed to identify peptides and assign modification localization. (**h**) Peptides are filtered for high confidence identification and modification site probability before downstream analysis. Created with BioRender.com.

The histone peptide microarray and K-OPL screen revealed that the antibodies most frequently used to enrich lysine methylation prior to MS analysis contain sequence bias. Less biased reagents are available but have not been implemented in an affinity purification-mass spectrometry (AP-MS) analysis (Fig. 2, Kme-2A compared to Kme-2D and Kme-3A compared to Kme3-E). We hypothesized that we could detect more sites by using less biased reagents (Kme2-D and Kme3-E). To compare the sites enriched by the two groups of antibodies, we performed a series of enrichments in two human cell lines, HEK293T and U2OS (Fig. 3). Mono-methyl lysine enrichment was conducted with the only commercially available mono-methyl antibody (Kme1-A) in every sample, thus resulting in two Kme1-A replicates. Next, the lysates were enriched for Kme2 with either the Kme2-A antibody or Kme2-D antibody. Finally, the Kme-3A antibody was used to enrich Kme3 following Kme2-A enrichment, and Kme3-E was used to enrich for Kme3 following Kme2-D enrichment. In a parallel experiment, two replicate enrichments were performed using Cell Signaling Technologies Pan-Methyl Lysine PTMScan kit, which contains Kme1-A, Kme2-A, and Kme3-A within a single mixture. This allows us also to compare the difference between multiple enrichments and a single enrichment using the same antibodies. A portion of each sample was also analyzed directly by LC–MS/MS without any enrichment to directly evaluate the efficiency of enrichment.

Following enrichment, we compared the unique lysine methylation sites enriched by the two antibodies for each methyl state (Kme2-A vs Kme2-D; Kme3-A vs Kme3-E). Kme2-A enrichment resulted in the identification of 17 Kme2 sites in U2OS and 50 Kme2 sites in HEK293T (Supplementary Fig. 2c). Kme2-D enrichment resulted in the detection of 39 sites in U2OS and 61 Kme2 sites in HEK293T. As expected, enrichment with the more pan Kme2-D antibody resulted in the detection of more Kme2 sites than Kme2-A enrichment in both HEK293T and U2OS cells. This trend was also observed for the Kme3 antibodies. Kme3-A enrichment resulted in the detection of 33 Kme3 sites in U2OS and 106 Kme3 sites in HEK293T, compared to 93 sites in U2OS and 114 sites in HEK293T following Kme3-E enrichment (Supplementary Fig. 2c). Interestingly, there was little overlap in sites enriched by the different antibodies.

Kapell et al. recently reported the reanalysis of a deep proteomics dataset for several human cell lines^27^. The inclusion of lysine methylation, arginine methylation, and histidine methylation resulted in the detection of hundreds of novel sites for each of these modifications. Their study highlights the potential to retrospectively analyze publicly available MS data and suggests new studies aiming to characterize lysine methylation may benefit from the analysis of fractionated lysates without any enrichment. Encouraged by these findings in the literature, we analyzed each of our fractionated samples by LC–MS/MS before and after enrichment so we could directly compare how affinity enrichment impacts the identification of lysine methylation sites.

**Figure 4:**
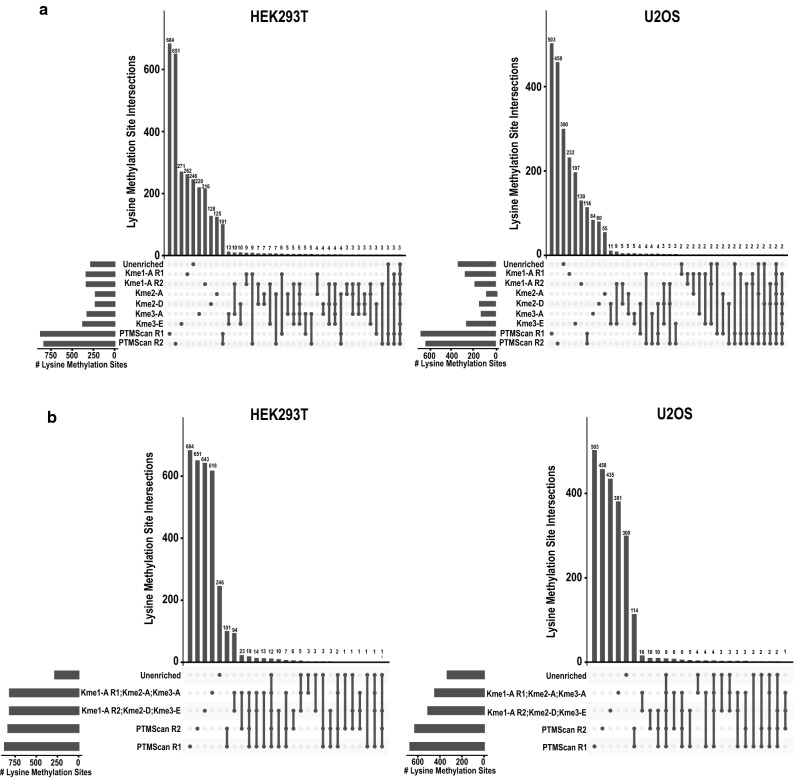
Lysine methylation enrichment impacts which lysine methyl sites are detected by mass spectrometry. (**a**) Upset plots showing lysine methylation sites identified using each enrichment strategy from HEK293T or U2OS lysates, as indicated. Horizontal bars on the left display the total number of lysine methylation sites identified by each enrichment strategy. Vertical lines connecting points represent overlap of identified sites. (**b**) Upset plots showing lysine methylation sites identified in unenriched samples, samples subjected to sequential antibody enrichment, or samples enriched with PTMScan kits from HEK293T or U2OS lysates, as indicated. R1 and R2 are replicates.

To assess whether enrichment was successful, we calculated the percentage of peptide spectral matches (PSMs) that contained a methylated lysine. For unenriched samples, this was below a fraction of a percent. In each of the enriched runs, greater than 5% of all PSMs contained a methylated lysine (Supplementary Fig. 3a). Furthermore, the highest percentage of PSMs in each enriched sample contained the methyl state that the antibody was directed against. This analysis suggests that enrichment, regardless of the antibody, was analytically effective. However, despite the lower percentage of PSMs in the unenriched samples containing lysine methylation, nearly as many unique lysine methylation sites were detected from the unenriched samples as any of the individual enrichments alone (Fig. 4a). Between the two PTMScan kit replicates, there is only ~ 20% overlap in sites detected, potentially highlighting the stochasticity of detecting lysine methylation. When comparing the number of lysine methylation sites identified either with individual runs combined by their sequential enrichments or with the PTMScan kit, however, substantially fewer sites were identified in the unenriched samples (Fig. 4b). Furthermore, there is about a 20% overlap in sites detected by the grouped enrichment strategies (PTMScan Rep 1 vs PTMScan Rep 2; Kme1A-Kme2A-Kme3A vs. Kme1A-Kme2D-Kme3E), while there is little overlap between the unenriched samples and any of the enrichment strategies, suggesting that an unenriched sample detects a different population of lysine methylation sites.

Overall, ~ 86% of all lysine methylation sites were only detected using a single enrichment strategy (Supplementary Fig. 3b) and less than 1% of lysine methylation sites were detected in at least half of all MS runs (Supplementary Table 4), reflecting minimal overlap between sites identified without enrichment or with different enrichment strategies. Overall, these data suggest that analysis of both unenriched and enriched samples may result in a more comprehensive profile of the lysine methylome.

### Impact of antibody sequence selectivity on identified lysine methylation sites

**Figure 5:**
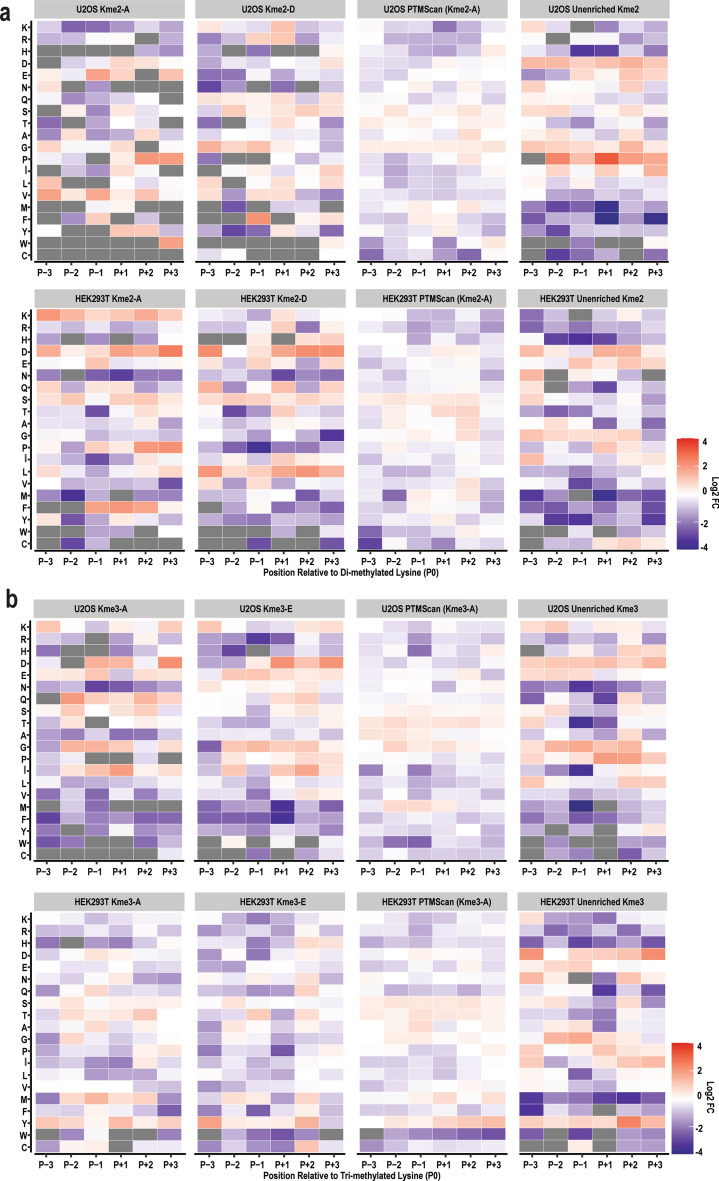
Comparison of sites detected by different pan-methyllysine antibodies. Heatmaps depicting the log_2_ of the ratio of the frequency of amino acids seen in the 7-mer motifs of methylated peptides compared to the frequency of amino acids seen in the 7-mer motifs of all lysine residues on every protein detected in unenriched samples. Gray squares indicate that the amino acid in that position was not detected on any peptide. (**a**) Di-methyl lysine peptides identified using enrichment with Kme2-A, Kme2-D, PTMScan, or no enrichment as indicated from U2OS or HEK293T cell lysates. (**b**) Tri-methyl lysine peptides identified following enrichment with Kme3-A, Kme3-E, PTMScan, or no enrichment as indicated from U2OS or HEK293T cell lysates.

We then sought to evaluate the sequence context surrounding lysine methylation sites identified by MS using different enrichment strategies. To this end, we first determined the 7-mer sequence motif surrounding every lysine in the human proteome and the 7-mer sequence motif of every lysine from all proteins detected in the unenriched samples. Notably, these were very similar. Overall, there is a higher frequency of K, E, S, L, and A in all positions, and H, M, F, Y, W, and C are the least frequent amino acids found near any lysine (Supplementary Fig. 4). Next, the frequency of observed amino acids near a methylated lysine was compared to the frequency in the corresponding proteome (Fig. 5) and compared to the motifs derived from K-OPL screening of each antibody.

Affinity purification from different antibodies resulted in the identification of peptides in a cell-type and enrichment-specific manner. Kme2-A strongly prefers D and E in the P+1 position according to K-OPL analysis (Fig. 2a), and this is reflected in di-methylated peptides detected in U2OS cells (Fig. 5a). In addition to D and E, di-methylated peptides from U2OS cells enriched with Kme2-A frequently contained Y. In contrast, while Kme2-A enriched peptides from HEK293T cells also had a higher frequency of D in the P+1 position, E was observed less frequently than expected. Additionally, instead of Y in the P+1 position, there was enrichment for K, S, and F. While some of the antibody selectivity preferences identified by K-OPL analysis were apparent in the peptides identified by MS, there were also discrepancies. We next sought to quantitatively assess whether the antibody bias detected by K-OPL analysis impacted the peptides enriched and identified by MS. A position-specific scoring matrix (PSSM) was calculated for the Kme2-A, Kme2-D, Kme3-A, and Kme3-E antibodies from the K-OPL microarray data. If the antibody bias detected by K-OPL analysis was skewing the identification of peptides by MS, the PSSM score for each identified peptide would be higher for the antibody used for enrichment. However, regardless of the enrichment strategy used, there was no difference in the average PSSM score for identified peptides (Supplementary Fig. 5).

The discrepancies in antibody selectivity captured by the K-OPL screening compared to MS identification of enriched peptides may be explained by several factors, including the sequence context of the peptides and the inherent nature of the assays^34–37^. Since trypsin cleaves after most lysine and arginine residues, it is possible that a large portion of the peptides identified may not have any amino acids on the C-terminus. While modifications on lysine typically block proteolysis, recent evidence suggest some modifications, including di-methyl lysine, may not completely block trypsin cleavage in all sequence contexts^34–37^. To address this possibility, we analyzed the location of the lysine methylation event in the context of the digested peptide and found that most peptides identified contained at least three amino acids on the N terminus and C terminus of the modified lysine residue (Supplementary Fig. 6). Other potential contributing factors could include the ability of the peptide microarray to capture antibody bias, either due to differences in epitope abundance (the K-OPL array presents the same concentration of all peptides and cell lysates present a non-uniform mixture of methylated peptides), limited sequence context of K-OPL peptides, structural preferences of antibodies, or differences in antibody binding to peptides immobilized on a surface versus in solution.

### The landscape of the expanded lysine methylome

**Figure 6:**
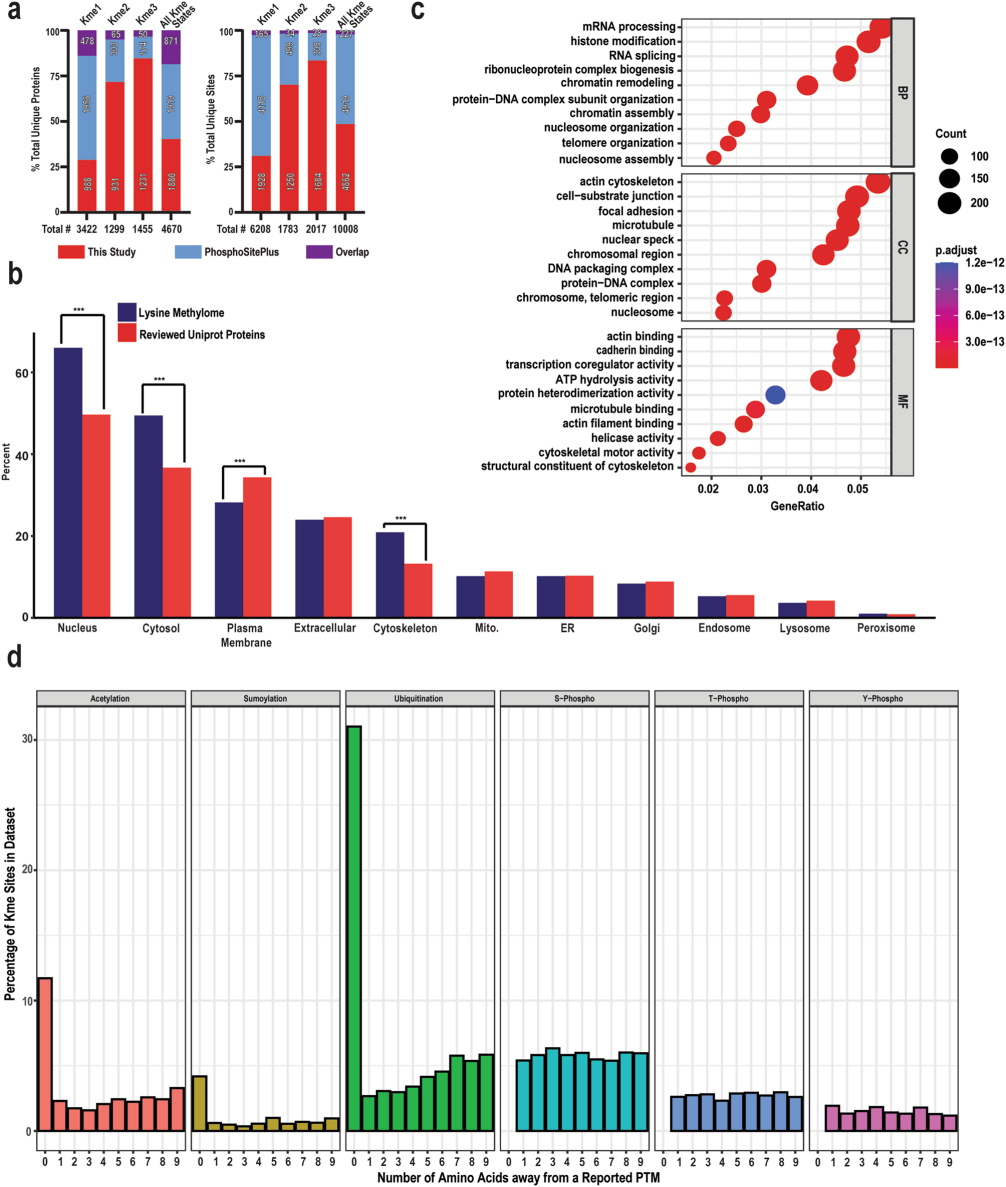
The landscape of the expanded lysine methylome. (**a**) Number of unique proteins and unique lysine methylation sites in the human lysine methylome and the percentage of sites identified in this study and those previously identified and recorded in the PhosphoSitePlus database. (**b**) Subcellular localization of all lysine methylated proteins compared to all human proteins (reviewed UniProt). (***) represents a p value < 2.0 e−16. (**c**) Over-representation analysis of all proteins with lysine methylation using the enrichGO function from the “clusterProfiler” package in R. Enriched ontologies—biological processes (“BP”), cellular compartments (“CC”), and molecular functions (“MF”)—identified using p. adjusted method of FDR with a cutoff of 0.05. (**d**) Analysis of the potential for PTM crosstalk. Percentage of Kme sites within 9 amino acids of another identified PTM (total sites, n = 10,413). All known acetylation, sumoylation, ubiquitination, and phosphorylation sites deposited from PhosphoSitePlus were used. Phosphorylation is subset by serine, threonine, and tyrosine sites.

Combined, this study resulted in the detection of 5089 unique lysine methylation sites on 2751 proteins. 4862 of these lysine methylation sites had not previously been reported in PhosphoSitePlus. A subset of these include 1880 proteins that had no previous reports of lysine methylation events in PhosphoSitePlus (Fig. 6; Supplementary Tables 5, 6). As expected, we identified canonical histone lysine methylation sites (H3K36me1/2/3, H3K27me1/2/3, H3K79 me1/2, and H4K20me2). In our experiments, the largest percentage gain of new lysine methylation sites were di-methyl and tri-methyl lysine (Fig. 6a). Over 70% of the Kme2 and Kme3 sites identified were novel, compared to just 28% for Kme1. Overall, after combining our data with the lysine methylation sites cataloged in the PhosphoSitePlus database, the lysine methylome consists of over 10,000 unique sites on 4670 unique proteins (Supplementary Table 7).

Lysine methylation sites identified in this study are evenly distributed throughout the proteome; ~ 91% of proteins with lysine methylation have one to three methylation events (Supplementary Fig. 7a). A subset of proteins have more lysine methylation events—some of which have previously reported lysine methylation sites, including AHNAK and AHNAK2^17^ (with 59 and 34 lysine methylation events, respectively), and some that have not been reported to have lysine methylation (Supplementary Table 6), such as Neurofilament heavy polypeptide (with 24 lysine methylation events) and Fibrous sheath-interacting protein 2 (with 16 lysine methylation events). We found no strong correlation (r^2^ = 0.1134) between the number of lysine methylation events per protein and number of lysine residues in amino acid sequences corresponding to identified methylated peptides (Supplementary Fig. 7b), suggesting that factors other than protein size, such as peptide abundance and sequence context surrounding methylated lysine, may be more influential in the effective identification of lysine methylation sites.

Given the large increase in detection of methylated proteins, we analyzed the combined 4670 methylated proteins to determine their localization and associated GO terms. For localization, each protein was assigned to a category based on the COMPARTMENT database^38^, revealing a significant enrichment in the percentage of methylated proteins found in the nucleus, cytosol, and cytoskeleton (Fig. 6b). Most other compartments showed no significant change, highlighting how widespread lysine methylation is within different cellular compartments. The only exception to this was a significant decrease in the percentage of proteins found in the plasma membrane. Gene ontology analysis identified enrichment for several biological processes related to RNA, including mRNA processing and RNA splicing (Fig. 6c). Other enriched processes included those expected to be associated with lysine methylation, including histone modification, chromatin remodeling, and nucleosome organization. The GO term actin cytoskeleton was among the top enriched in the cellular compartment category, in alignment with the enrichment of methylated proteins in the cytoskeleton. This analysis highlights the diversity of cellular processes lysine methylation may regulate and provides a roadmap to explore the function of lysine methylation in future studies.

One of the major functions of lysine methylation on both histone and non-histone proteins is to mediate crosstalk with other PTMs to regulate cellular signaling. To determine the potential for crosstalk among the entirety of the lysine methylome, we calculated the percentage of lysine methylation sites in the lysine methylome near a known acetylated, sumoylated, ubiquitylated, or phosphorylated site at each position within nine amino acids of the methylated lysine using data from the PhosphoSitePlus (Fig. 6d)^28^. Ubiquitylation and acetylation are most frequently directly competing with methylation, often occurring at the same amino acid site where methylation occurs. In contrast, phosphorylation is equally distributed across all amino acids 1–9 positions away from a methylated lysine. Of note, serine phosphorylation is found more often than either threonine or tyrosine phosphorylation, consistent with the increased frequency of serine in the lysine methylome (Supplementary Fig. 4). Overall acetylation, sumoylation, ubiquitylation, or phosphorylation has been observed within nine amino acids of 66% of identified lysine methylation sites. Within our own study, we observed a co-occurrence of lysine methylation and phosphorylation on around ~ 15 to 25% of all methylated peptides regardless of the enrichment strategy (Supplementary Fig. 8). Future studies are required to directly characterize the co-occurrence or mutual exclusivity of other PTMs with lysine methylation.

## Discussion

In this study, we have systematically characterized the selectivity of pan-methyllysine affinity reagents and compared their performance in LC–MS/MS workflows for global profiling of lysine methylation. Our experiments revealed that most of the affinity reagents for lysine methylation display sequence bias that impacts sites detected when used in global lysine methylation profiling. The selectivity profiles for each of these reagents will be a critical resource to inform future studies. The use of oriented peptide libraries had previously been used for pan-antibody characterization^39^, and the work presented herein provides a useful framework for future characterization of pan antibodies that recognize other PTMs.

Despite progress, proteome-wide mapping of lysine methylation remains challenging and has not been implemented in many tissue or cell types. A recent study predicted that nearly 50,000 unique lysine methylation sites may exist on human proteins, suggesting the lysine methylome may be at least an order of magnitude larger than the sites observed thus far^40^. This study results in a nearly 50% increase in the number of unique lysine methylation sites on human proteins, and future studies in additional cell types may bring the final number closer to what has been predicted.

Another recent study reanalyzed tandem MS data and was able to identify novel lysine methylation sites from deeply fractionated human cell lysates without affinity enrichment^27^. In our work, we build upon this report to directly compare lysine methylome profiling with or without enrichment. We conclude that both strategies lead to the identification of novel lysine methylation sites with little overlap, and in future studies aimed at characterizing lysine methylation proteome-wide, it may be beneficial to analyze samples using both deep fractionation and affinity enrichment.

KMTs and KDMs are dysregulated in many diseases and dynamically expressed in different cell and tissue types. As the field moves beyond histone proteins, quantitatively comparing non-histone lysine methylomes in different biological or disease contexts is a requirement for understanding the function of these enzymes. In our study, affinity enrichment resulted in a greater number of identified methylation sites, but it also requires expensive affinity reagents. Another important consideration is the sample material requirements, which severely limit the type of quantitative workflows and samples that can be analyzed. The ability to identify lysine methylation proteome-wide sans-affinity enrichment enables quantitative analysis of lysine methylation using MS workflows, such as tandem mass tags, that were previously cost prohibitive with the sample material required for each enrichment step. In the future, quantitative analysis of lysine methylation changes will be important to further our understanding of the role that KMTs and KDMs play in different disease and developmental contexts.

## Methods

### Peptide microarrays

Peptide microarrays were designed using ArrayNinja^41^ and fabricated using an Aushon 2470 microarrayer as described previously^26^. Antibodies were diluted in Array Hybridization Buffer (137 mM NaCl, 2.7 mM KCl, 10 mM Na_2_HPO_4_, 1.8 mM KH_2_PO_4_, pH 7.6, 5% BSA, 0.1% Tween-20) and incubated over each microarray slide for 1 h at 4 °C. Slides were washed in PBS, incubated with fluorescently labeled secondary antibodies (Life Technologies A-21244 or A-21235), washed in PBS, and scanned using an Innopsys InnoScan 1100AL microarray slide scanner. Microarray images were analyzed using ArrayNinja software.

### Cell culture

HEK293T and U2OS cells were cultured in Dulbecco’s Modified Eagle Media (Corning) supplemented with 10% (v/v) fetal bovine serum (Sigma) and 1 × Penicillin/Streptomycin (Corning). Cells were grown in 15 cm dishes and collected when they reached ~ 80% confluency. Pellets were washed in PBS and stored at −80 °C.

### Proteomics sample preparation

Cell pellets were resuspended in urea lysis buffer (8 M urea, 50 mM Tris–HCL pH 8.5, 100 U of benzonase) and quantified by Bradford assay. A total of 30 mg of total protein lysate for each cell line were reduced with 5 mM tris(2-carboxyethyl)phosphine hydrochloride, alkylated with 10 mm chloroacetamide, and digested overnight at room temperature with trypsin/lysC (1:200 ratio protease: substrate, Promega cat no: V5072). Digestion reactions were quenched by adding 0.2% trifluoroacetic acid (TFA) and desalted using three 500 mg SepPak vacuum manifold (loading approximately 10 mg per cartridge) with washes of 5 mL 0.1% TFA and elution in 70% acetonitrile, 0.1% TFA. Peptides were lyophilized and resuspended in 1 mL of 10 mM formate, 5% acetonitrile, pH 10 and centrifuged for 10 min at 12,000*g* prior to offline high pH basic fractionation.

Peptides were fractionated using an Ultimate 3000 HPLC (Thermo Fisher Scientific™) with a Waters Xbridge^®^BEH C18 OBD™ Prep column (130A, 5 um, 10 mm × 250 mm, Part No 186008167) run at 2.4 mL/min in Solvent A (10 mM formate 5% acetonitrile, pH 10). The gradient was held at 5% B for 3 min, 5–15% B in 2 min, 15–20% B in 5 min, 20–35 over 45 min, 35–50% B over 15 min, and 50–70 over 5 min before being held at 70% B for 5 min with continuous 2 mL fractions being collected throughout. Solvent B consist of 100% acetonitrile and 0.1% FA. Fractions were concatenated sequentially back down to 5 total fractions and lyophilized. A portion of each fractionated sample was analyzed independently as “global peptides” while the remainder was used for enrichment of methylated peptides.

### Enrichment of methylated peptides

Peptides fractions were resuspended in 1 mL PBS and the pH was determined by spotting a small amount of each fraction onto a pH strip to ensure the pH was neutral. For each fraction, 200 µL of Protein A Mag Sepharose beads (GE) were washed with PBS for a total of three washes followed by resuspension in 400 µL of antibody and incubated for 4 h at 4 °C on a rotator. The resulting bead-antibody complex was washed with 1 mL of PBS for a total of three washes. After the last wash, the bead complex was resuspended with 1 mL of peptides and incubated overnight at 4 °C on a rotator. The supernatant was collected and saved for subsequent enrichment. The beads were washed by rotating with 1 mL 0.8 M NaCl for five minutes at room temperature for a total of three washes. The beads were washed by rotating with 1 mL of water for five minutes at room temperature, transferred into a new microtube, followed by two additional washes with water. To elute the peptides, the beads were resuspended in 100 µL of 0.1% TFA and incubated for 10 min at room temperature on a vortex on the low setting. The supernatant was collected and pooled with the supernatant from two additional elutions. Eluted peptides were dried in a speed vacuum and resuspended in 20 µL 0.1% FA and centrifuged at 14000*g* for 20 min prior to LC–MS/MS.

### LC–MS/MS of enriched peptides

Each fraction of enriched peptides was run in technical replicate, injecting approximately half each time. Peptides were separated on an Ultimate 3000 HPLC with loading on a 5 cm C18 trap column Acclaim™ PepMap™ 100 (3 µm particle size, 75 µm diameter; Thermo Scientific, Cat No: 164946) followed by a 15 cm PepMap RSLC C18 EASY-Spray column (Thermo Scientific, Cat No: ES900) and analyzed using a Q-Exactive Plus mass spectrometer (Thermo Fisher Scientific) operated in positive ion mode. After loading the trap column at 3 µL/min for 7 min, the column was switched inline and at 400 nL/min peptides were separated 5–35% solvent B over 60 min, 8–50% solvent B over 30 min, 50–95% solvent B over 1 min, held at 95%B for 2 min and 95–8% B over 1 min (Solvent A: 95% water, 5% acetonitrile, 0.1% formic acid; Solvent B: 100% acetonitrile, 0.1% formic acid). A data-dependent TopN20 mass acquisition method was used with the following parameters for the MS1: mass resolution: 70,000; AGC target: 3e6; maximum IT: 100 ms; and mass scan range: 300–2000 m/z. MS2 parameters were fixed first mass: 100 m/z; resolution: 17,500; AGC target: 1e5; maximum IT: 50 ms; isolation window: 4.0 m/z; normalized collision energy: 30; dynamic exclusion: 30 s; and charge exclusion: 1, 7, 8, > 8.

### LC–MS/MS of global peptides

Global proteomics nano-LC–MS/MS analyses were performed on an EASY-nLC 1200 HPLC system (SCR: 014993, Thermo Fisher Scientific) coupled to Exploris 480™ mass spectrometer (Thermo Fisher Scientific). Approximately 2 µg of each global peptide fraction was separated on a 25 cm EasySpray™ C18 column (2 μm, 100 Å, 75 μm × 25 cm, Thermo Scientific Cat No: ES902A) at 400 nL/min. Peptides were eluted from 4 to 30% with mobile phase B over 160 min, 30–80% B over 10 min; and dropping from 80 to 10% B over the final 10 min. The mass spectrometer was operated in positive ion mode with FAIMS CVs of − 40, − 55 and − 70 (1.3 s cycle time per CV with identical precursor and MS/MS scan parameters), data dependent acquisition method with advanced peak determination and lock mass of 445.12003. Precursor scans (m/z 375–1500) were done with an orbitrap resolution of 120,000, RF lens% 40, maximum inject time auto, standard AGC target, MS2 intensity threshold of 5e3, MIPS mode, including charges of 2–7 for fragmentation with 30 s dynamic exclusion shared with all scans. MS2 scans were performed with a quadrupole isolation window of 1.6 m/z, 28% HCD CE, 15,000 resolution, standard normalized AGC target, auto maximum IT, fixed first mass of 110 m/z.

### Proteomics data analysis

Raw files were analyzed with Proteome Discoverer 2.5.04.400 (Thermo Fisher Scientific™) with a Uniprot database of reviewed *Homo sapiens* proteins downloaded 092929 with common laboratory contaminants (20350 sequences total). For enriched samples, Sequest HT was used with full trypsin specificity, a max of 5 missed cleavage sites, precursor tolerance of 10 ppm, fragment tolerance of 0.02 Da. The static modification used was carbamidomethyl of C, and the dynamic modifications included were oxidation of M, methylation of K, dimethylation of K, trimethylation of K, phosphorylation of S, T, Y and protein N terminus acetylation or Met-loss. Peptide spectral match (PSM) false discovery rate was dictated by Fixed Value PSM validator (max delta Cn of 0.05 from SEQUEST HT), and Imp-PTMRS node was used for localization of methyl and phospho modifications. For unenriched samples, the same search conditions were used, but Percolator was used as the FDR node with target/decoy concatenated selection with q value validation and strict FDR of 0.01.

### Subcellular localization analysis

The Uniprot ID entries for all the reviewed proteins were downloaded from Uniprot.org (downloaded January 2022). Cytoscape v.3.9.0 stringAPP v.1.7.0 was used to determine protein subcellular localization. All subcellular compartments with a score of 3 or greater for a given protein were considered to be the compartments to which an individual protein localized. The proportion of proteins found in each compartment was calculated for the human lysine methylome and all reviewed Uniprot proteins. A 2-proportion Z-test was used to determine statistical significance between the two proportions for each compartment.

### Bioinformatic analysis

*Gene Set enrichment analysis*: R package ClusterGo was used to determine the categorical annotations of protein Gene Ontology (GO) biological process, cellular components, and molecular function. *Sequence motif analysis*: sequences were extracted from Uniprot using the R package UniprotR. For both the lysine-centric human proteome and the underlying proteome, the frequency of an amino acid in each position was determined. The underlying proteomes for each dataset were calculated by pooling all lysine residues on proteins identified in the unenriched experiments specific to that cell type. To calculate the motif of each dataset, the following equation was used: $log2\frac{{f}^{d}}{{f}^{p}}$, where ${f}^{d}$ is the frequency of an amino acid in each position in the dataset and ${f}^{p}$ is the frequency of an amino acid in the underlying proteome. Heatmaps were made using the R package ggplot2. *Position-specific score matrix (PSSM) scores*: PSSM scores for each antibody were calculated from the K-OPL selectivity data, where each amino acid in a specific position has a frequency score (Supplemental Table 3). The frequency scores of the amino acid were summed for each individual 7-mer peptide with a central lysine in the human proteome, thereby giving a score for all lysine centralized 7-mer peptides in the human proteome. The output is a ranked list of preferred peptides from each of the 10 antibodies based on the K-OPL data.

## Supplementary Information

Supplementary Information 1.

Supplementary Information 2.

Supplementary Information 3.

## Data Availability

The datasets generated from the current study are available in the MassIVE repository, under Accession Number MSV000090519: https://massive.ucsd.edu/ProteoSAFe/dataset.jsp?task=040dc093a1bc4cbdbcb879ec733f7efc.
