# Supplementary material for: Global lysine methylome profiling using systematically characterized affinity reagents
Source: Sci Rep. 2023 Jan 7;13:377. doi: 10.1038/s41598-022-27175-x (PMC9825382; doi:10.1038/s41598-022-27175-x)
Supplement: Supplementary file 1 — Supplementary Information 1. [file 41598_2022_27175_MOESM1_ESM.pdf]

Supplementary Materials for

Global Lysine Methylome Profiling Using Systematically Characterized Affinity Reagents

Christine A. Berryhill[1,3], Jocelyne N. Hanquier[1,3], Emma H. Doud[1], Eric Cordeiro-Spinetti[2], Bradley M. Dickson[2], Scott B. Rothbart[2], Amber L. Mosley[1], Evan M. Cornett[1*]

[1]Department of Biochemistry and Molecular Biology, Indiana University School of Medicine, Indianapolis, IN 46202, U.S.A.[2]Department of Epigenetics, Van Andel Institute, Grand Rapids, MI 49503, U.S.A. [3]These authors contributed equally: Christine A. Berryhill and Jocelyne N. Hanquier

[*]To whom correspondence should be addresses: evcorn@iu.edu

The PDF file includes:

Supplementary Table 1: Antibodies used in this study.

Supplementary Fig. 1: Schematic for analysis of pan-Kme2 and Kme3 antibodies using lysine-oriented peptide microarrays.

Supplementary Fig. 2: Evaluation of the “pan”-ness of pan-methyllysine antibodies.

Supplementary Fig. 3: Efficiency of enrichment.

Supplementary Fig. 4: Motifs of the lysine methylome and lysine-oriented proteome.

Supplementary Fig. 5: Relationship between the 7-mer peptide PSSM score and enrichment strategy.

Supplementary Fig. 6: Position of methylated lysines in detected peptide fragments.

Supplementary Fig. 7: There is no relationship between the number of lysine methylation events per protein and protein size.

Supplementary Fig. 8: Co-occurrence of phosphorylation and lysine methylation on all methylated peptides

Other Supplementary Material includes the following:

Supplementary Table 2: Peptides on histone peptide microarrays.

Supplementary Table 3: K-OPL array data.

Supplementary Table 4: Prototypical non-histone Kme sites frequently identified.

Supplementary Table 5: All lysine methylation sites identified in this study.

Supplementary Table 6: Newly identified methylated proteins.

Supplementary Table 7: Combined lysine methylome containing data from this study and PhosphoSitePlus.

Supplementary Table 1: Antibodies used in this study.

Code	Company	Catalog	Lot
Kme1-A	Cell Signaling Technology	14679S	2
Kme2-A	Cell Signaling Technology	14117S	1
Kme2-B	Abcam	ab7315	GR3185886-1
Kme2-C	PTM Biolabs	PTM602	PTJD009P1
Kme2-D	PTM Biolabs	PTM606	ZD068E922P4
Kme3-A	Cell Signaling Technology	14680S	1
Kme3-B	Abcam	ab23366	GR32966
Kme3-C	Novus	NBOP1-78104	30589
Kme3-D	Abcam	ab76118	
Kme3-E	PTM Biolabs	PTM601	ZD151G017P4

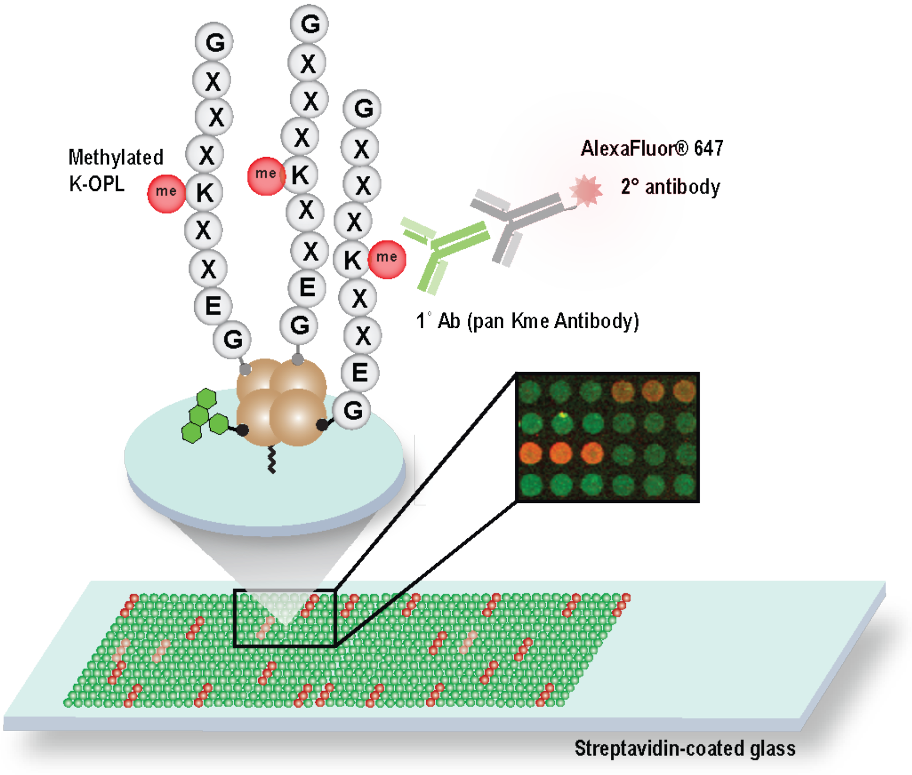

Supplementary Figure 1. Schematic for analysis of pan-Kme2 and Kme3 antibodies using lysine-oriented peptide microarrays. Schematic of microarray analysis of antibody selectivity using lysine-oriented peptide libraries (K-OPL). C-terminally biotinylated K-OPLs containing a central unmodified, mono-, di-, and tri-methylated central lysine were immobilized on a streptavidin-coated glass microarray slide. Pan-methyllysine antibodies were incubated on the surface of the slide followed by detection using a fluorescent secondary antibody.

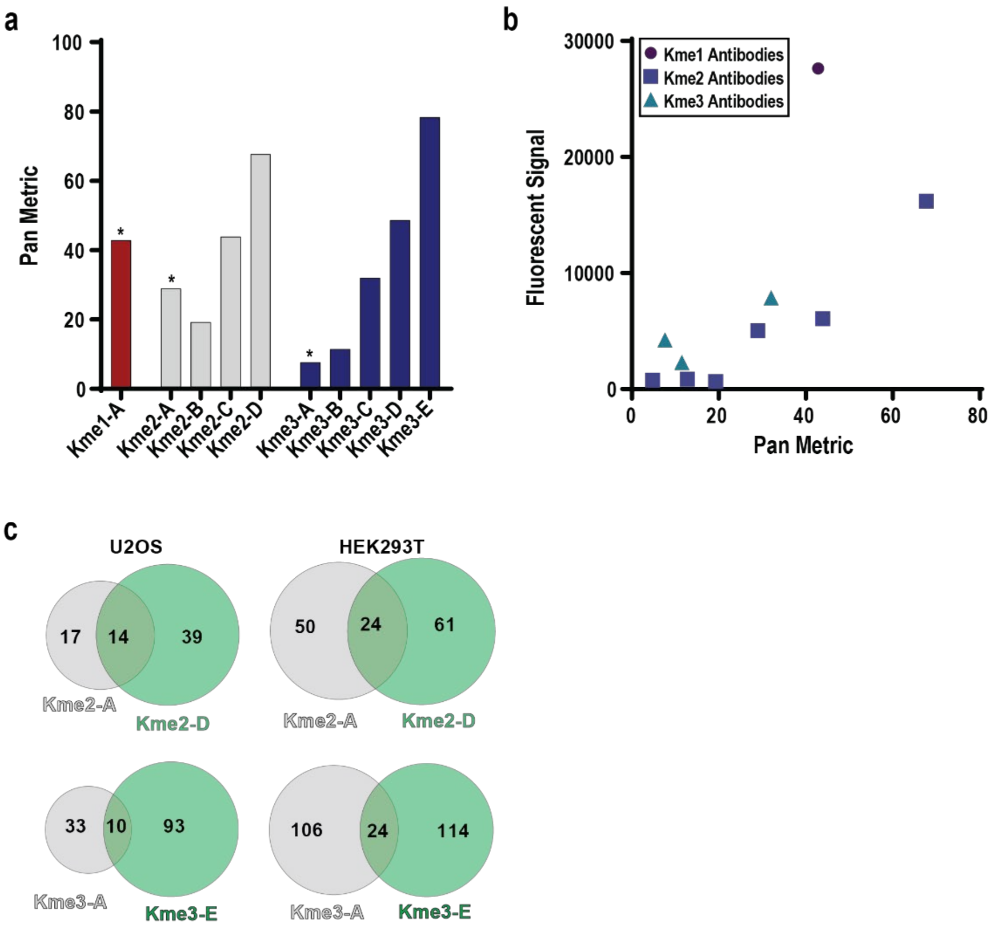

Supplementary Figure 2. Evaluation of the "pan"-ness of pan-methyllysine antibodies. a) Using the lysine-oriented peptide microarray data for the intended lysine methyl state of each antibody, a "pan" metric was calculated. After normalizing all signals to the highest feature on the array, all values were summed, divided by the number of features, and multiplied by 100. Thus, a pan metric value of 100 would have equal signal across every feature on the microarray. Asterisks represent commercially available antibodies most frequently used in the literature to enrich for lysine methylation before MS. **b)** Relationship between fluorescent intensity and the pan metric score. Fluorescent intensity is the highest value observed. **c)** Venn diagrams showing overlap of di-methyl or tri-methyl lysine sites identified by mass spectrometry following enrichment with the indicated antibodies from U2OS or HEK293T cell lysates.

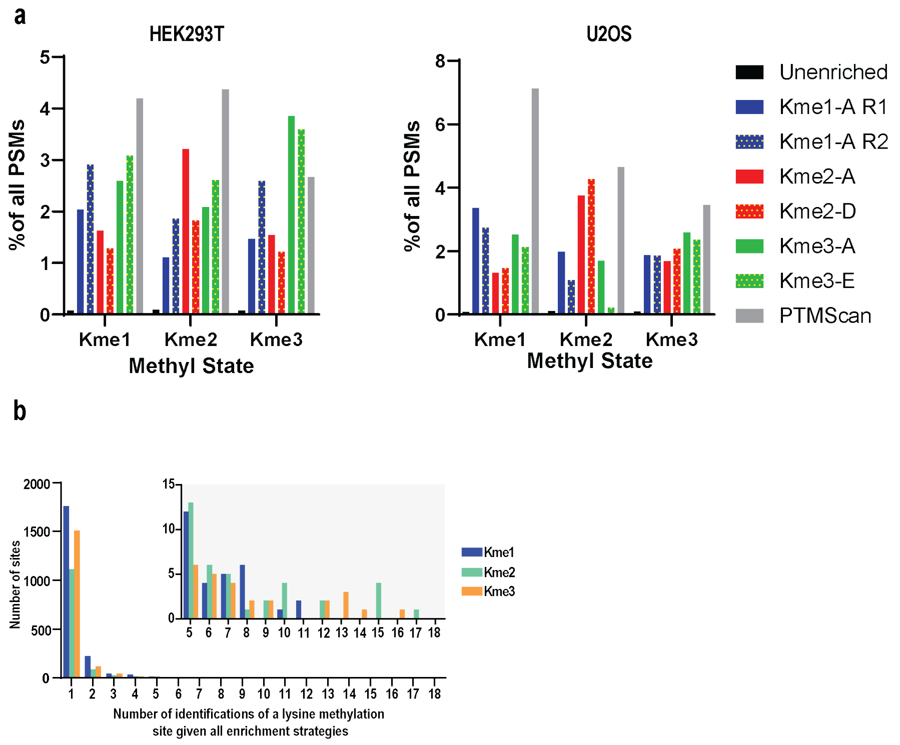

Supplementary Figure 3. Efficiency of Enrichment. a) Percentage of peptide spectral matches (PSMs) containing lysine methylation from HEK293T or U2OS cells based on enrichment strategy as indicated. **b).** Distribution of identification frequency for methylation sites from all LC-MS/MS experiments in this study (possible total of 18 identifications, given 9 enrichments and one run without antibody enrichment in two cell lines).

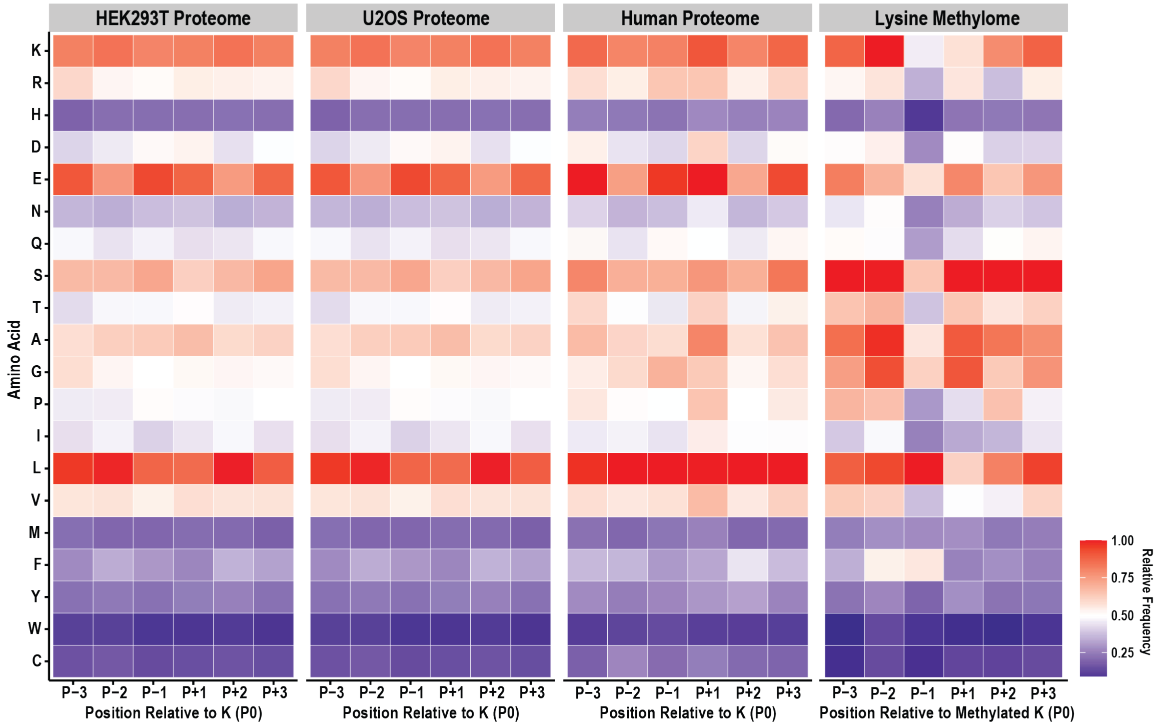

Supplementary Figure 4. Motifs of the lysine methylome and lysine-oriented proteome. Heatmaps depicting the frequency of amino acids ±3 residues from an unmethylated lysine from global proteomics of HEK293T, U2OS, all reviewed proteins in UniProt, or methylated lysine from the combined lysine methylome consisting of data from this study and the PhosphoSitePlus database.

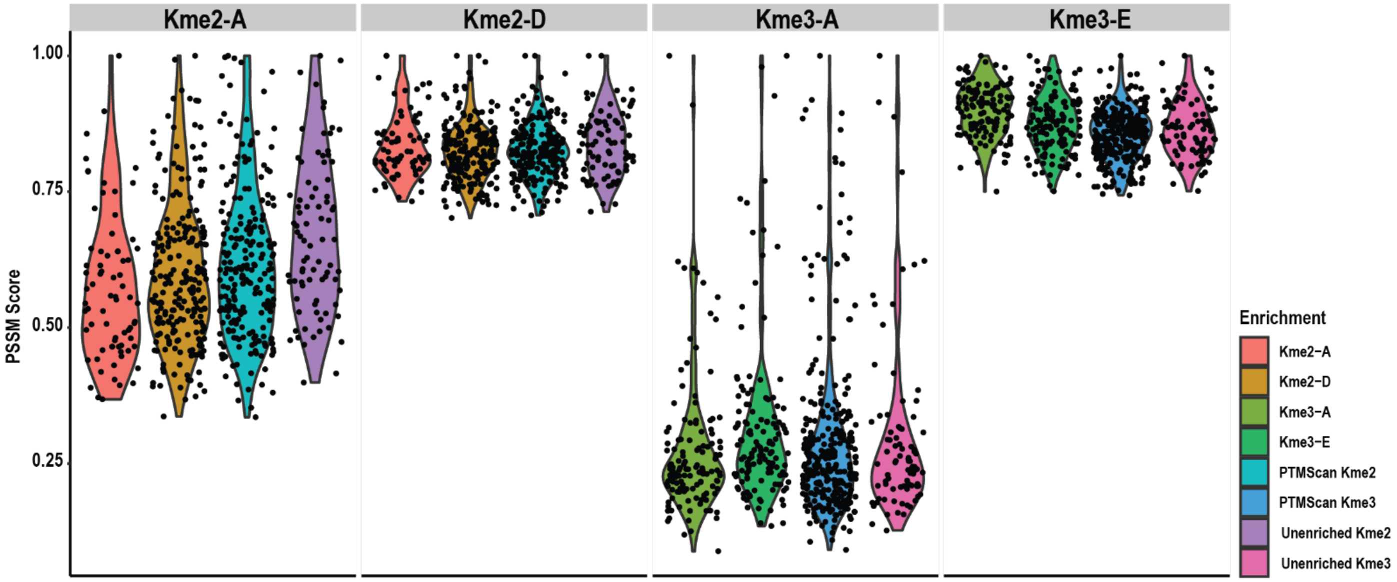

Supplementary Figure 5. **Relationship between the 7-mer peptide PSSM score and enrichment strategy.** PSSM scores were calculated using the K-OPL data for each methylated peptide identified in each MS enrichment strategy of a specific state (either di- or tri- methyl) (Supplementary Figure 2). The grouped PSSM scores (Kme2-A, Kme2-D, Kme3-A, or Kme3-D scores) were normalized to the highest score. Top labels indicate the antibody PSSM group and colors indicate the enrichment.

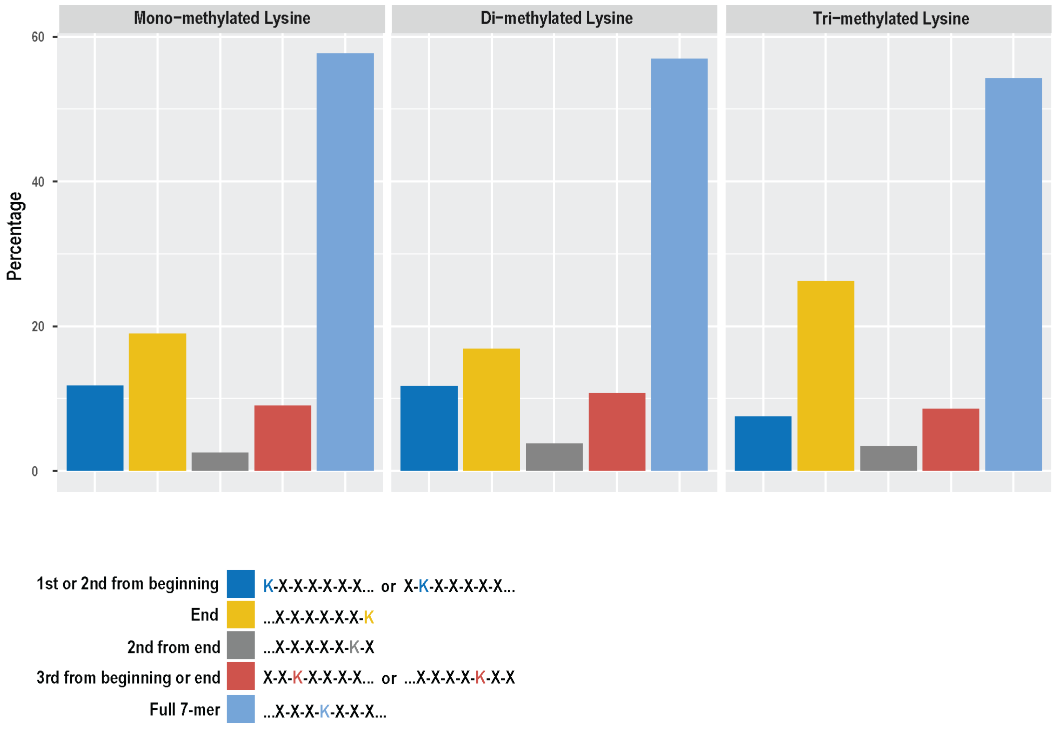

Supplementary Figure 6. Position of methylated lysines in detected peptide fragments. Percentage of detected methylated lysines in all MS runs conducted in this study at either the beginning or the 2nd residue of the peptide (dark blue), the end of the peptide (yellow), 2nd residue from the end of the peptide (gray), 3rd residue from either the beginning or end of the peptide (red), or with the full 7-mer present in the peptide (light blue). Data is separated by the detected methyl state on the peptide.

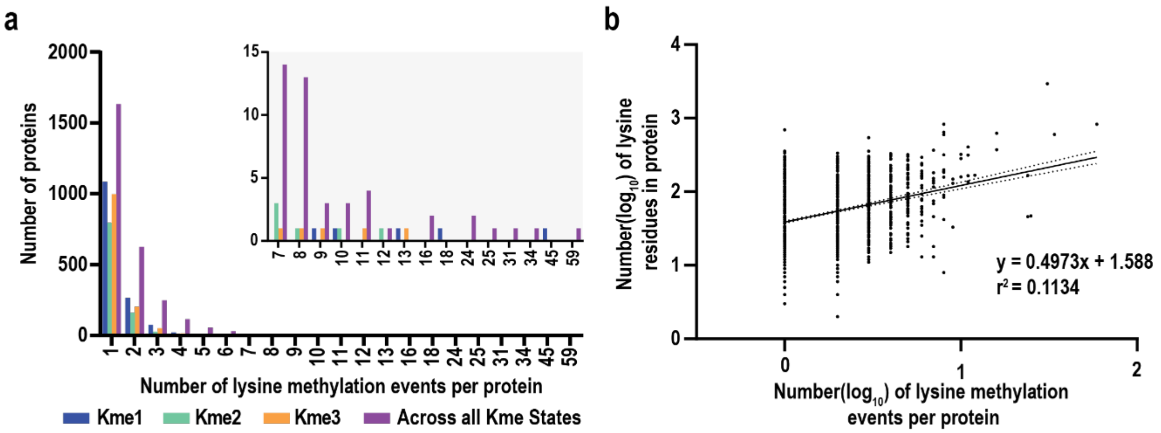

Supplementary Figure 7. There is no relationship between the number of lysine methylation events per protein and protein size. **a).** Number of lysine methylation events per protein for lysine methylation sites identified from HEK293T and U2OS cells. **b).** Comparison of the number of lysine methylation events per protein from HEK293T and U2OS cells with the number of lysine residues in each protein. The graph displays the best fit line from linear regression; correlation analysis reveals a poor correlation ($r^2 = 0.1134$) between the number of lysine methylation sites and the number of lysine residues.

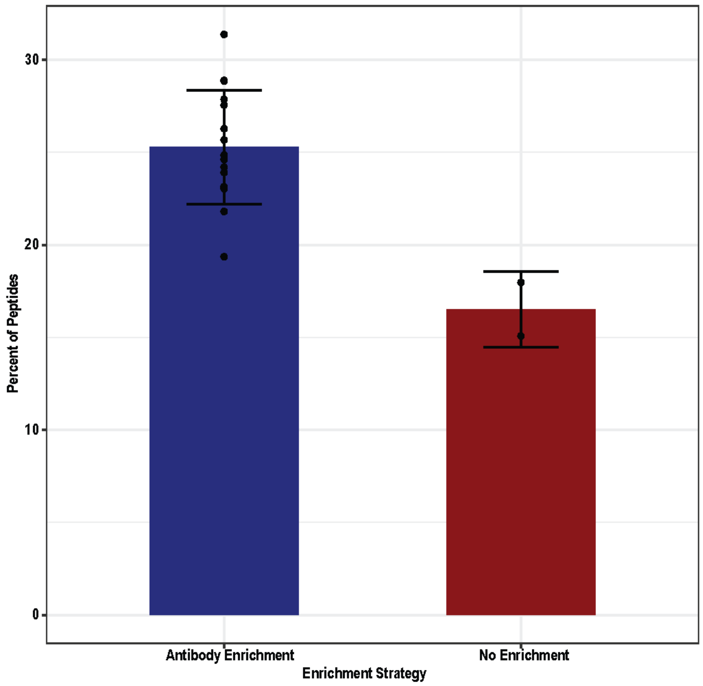

Supplementary Figure 8. Co-occurrence of phosphorylation and lysine methylation on all methylated peptides. Percentage of methylated peptides that also contain a phosphorylated residue, separated by enrichment strategy. Each point represents a different MS run.
